# Comparison of Antimicrobial Efficacy of Diode Laser, Triphala, and Sodium Hypochlorite in Primary Root Canals: A Randomized Controlled Trial

**DOI:** 10.5005/jp-journals-10005-1399

**Published:** 2017-02-27

**Authors:** Seby Thomas, Sharath Asokan, Baby John, Geetha Priya, S Kumar

**Affiliations:** 1 Postgraduate Student, Department of Pedodontics, K.S.R. Institute of Dental Science & Research, Tiruchengode, Tamil Nadu, India; 2 Professor, Department of Pedodontia, K.S.R. Institute of Dental Science & Research, Tiruchengode, Tamil Nadu, India; 3 Professor and Head, Department of Pedodontia, K.S.R. Institute of Dental Science & Research, Tiruchengode, Tamil Nadu, India; 4 Reader, Department of Pedodontia, K.S.R. Institute of Dental Science & Research, Tiruchengode, Tamil Nadu, India; 5 Postgraduate Student, Department of Endodontics, K.S.R. Institute of Dental Science & Research, Tiruchengode, Tamil Nadu, India

**Keywords:** Laser, Sodium hypochlorite, Triphala.

## Abstract

**Aim:**

To evaluate the antimicrobial efficacy of diode laser, triphala, and sodium hypochlorite (NaOCl) against *Enterococc-cus faecalis* contaminated primary root canals.

**Materials and methods:**

Forty-nine single-rooted human primary teeth were reduced up to cemento-enamel junction and biomechanically prepared. After sterilization, five teeth were selected as negative controls and remaining teeth were inoculated with *E. faecalis.* The teeth were then randomly divided into four groups. The first group was irradiated with diode laser, the second group was irrigated with sodium hypochlorite, and the third group with triphala solution. The fourth group served as the positive control. The antimicrobial efficacy was tested by collecting transfer fluid saline from the canals and counting the colony forming units (CFUs) of viable *E. faecalis* on agar plates. The Mann-Whitney test was used to analyze the results, using Statistical Package for the Social Sciences software version 19.

**Results:**

The results showed that mean bacterial CFU were 8.00 ± 7.87 for laser, 58.60 ± 16.63 for triphala, and 69.80 ± 19.57 for NaOCl. Laser group showed significant reduction in the colony count compared to the other groups. Triphala group showed better antibacterial activity than NaOCl, but the difference was not statistically significant.

**Conclusion:**

Laser was most effective against *E. faecalis* and triphala can be used as an alternative disinfectant to NaOCl in primary root canals.

**How to cite this article:**

Thomas S, Asokan S, John B, Priya G, Kumar S. Comparison of Antimicrobial Efficacy of Diode Laser, Triphala, and Sodium Hypochlorite in Primary Root Canals: A Randomized Controlled Trial. Int J Clin Pediatr Dent 2017;10(1):14-17.

## INTRODUCTION

Pulpectomy involves complete removal of necrotic pulp tissue in a primary tooth. The main goal of pulpectomy is to disinfect the root canal completely. The biomechanical preparation in primary teeth is done, not only to enlarge the pulp canals but also to remove pulpal tissues. It is impossible to remove all the remaining tissue due to the tortuous root canals in the primary teeth. Use of mechanical instrumentation alone cannot clean this tubular network sufficiently. So irrigants have been used along with mechanical instrumentation to achieve better debridement. Medicaments are used to improve the prognosis and predictability of endodontic treatment. They are used to eliminate any remaining viable bacteria in the root canal system that has not been destroyed by biomechanical preparation. It reduces periradicular inflammation, prevents or arrests inflammatory root resorption, prevents reinfection of the root canal system, and reduces pain.^1^

*Enterococcus faecalis* is a facultative organism which is resistant to various intracanal medicaments and persistently found in infected primary root canals.^2^ Sodium hypochlorite (NaOCl) and chlorhexidine (CHX) have shown antibacterial activity against *E. faecalis.* But studies by Estrela et al,^3^ showed that NaOCl or CHX have low ability to reduce *E. faecalis.* have demonstrated good antimicrobial action in the root canal system. Gutknecht et al^4^ showed that diode laser had reduced 74% of *E. faecalis* under *in vitro* conditions. Diode laser showed good results in *in vivo* conditions also. It could eliminate the microorganisms by deep penetration into the dentinal tubules.^5^

Over the last decade, use of herbal drugs as intracanal irrigants in endodontic treatment has increased. Triphala is an ayurvedic medicine that mainly contains “halituki” *(Terminalia chebula),* “amulaki” *(Emblica officinalis),* and “bibhitaki” *(terminalia bellirica).* The citric acids from the fruits aid in the removal of smear layer and act as chelat-ing agent. Prabhakar et al^6^ evaluated the antimicrobial activity of triphala against *E. faecalis* in permanent teeth and have suggested it as an alternative to NaOCl. Literature search showed no articles on the use of triphala as an intra canal irrigant in primary root canals. Hence this study was planned to evaluate and compare the antimicrobial efficacy of triphala with the gold standard sodium hypochlorite and lasers in primary root canals contaminated with *E. faecalis.*

**Table 1:** **Table 1:** Mean bacterial colony forming units in different groups

		*Positive control*		*Negative control*		*NaOCl*		*Laser*		*Triphala*	
Mean ± standard deviation		295.40 ± 21.90		0		69.80 ± 19.57		8.00 ± 7.87		58.60 ± 16.63	
Median		293.00		0		71.00		8.00		64.00	

## MATERIALS AND METHODS

The study protocol was analyzed and approved by the Institutional Review Board of K.S.R. Institute of Dental Science and Research, Tiruchengode, Tamil Nadu, India. The study comprised of 49 single-rooted human primary teeth which did not have physiological or pathological resorption more than apical %rd of root. The crowns of the teeth were reduced up to the cement enamel junction. Access opening was done and the pulp was removed with a barbed broach, and the root canals were prepared using stainless steel K files up to 40 size. Sterile saline solution was used as an irrigant, and the root canals were dried using sterile paper points. The apical foramina were sealed with composite resin. The samples were then autoclaved for 15 minutes at 121°C. Five teeth were selected randomly to serve as negative controls with no bacterial contamination. A pure culture of *Enterococous faecalis* in nutrient broth was used for inoculation into the remaining 44 teeth. A 0.01 ml suspension from this nutrient broth was inoculated into each canal using a sterile insulin syringe. Then samples were incubated for one week under aerobic conditions. To provide sufficient nutrients, 0.01 ml of nutrient broth was added every day into each canal for one week and incubated. After the incubation period, five inoculated teeth were chosen to serve as positive controls, while the rest were randomly divided into three experimental groups of 13 canals each. Randomization was done using a computer generated table of random numbers. The nutrient broth inside the canal was dried out completely, using sterile paper points before intervening each canal. In first group laser irradiation was done five times for 5 seconds, with a 15-second interval between the irradiations. Laser treatment was carried out with a diode laser (Piccaso, AMD laser, Dentsply, USA), at a wavelength of 810 nm and output power of 2 Watts (W) in the repeated pulse mode, using a pulse duration of 20 minutes and a pulse interval of 40 minutes. Irradiation was performed with circling movements from down to up motion. The second group was irrigated with 3% NaOCl solution (Vensons India, Banglaore) for 5 minutes. The third group was irrigated with triphala solution [made by dissolving the commercially available triphala powder (Nilogram India Pvt. Ltd, Bangalore) in the solvent 10% dimethyl sulfoxide (DMSO) in 1:3 dilution] for 5 minutes. In positive control group five teeth were irrigated with 5 mL of normal saline for 5 minutes.

**Figs 1A to C:**
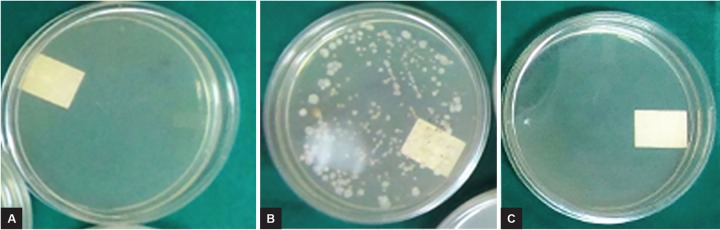
Sample of nutrient agar plate: (A) After spreading the transfer fluid from root canals; (B) after incubation with numerous colonies of *E. faecalis;* and (C) after incubation with few colonies of *E. faecalis*

The root canals of all the teeth were dried with sterile paper points, after the completion of procedures. The canals of all the teeth were refilled with normal saline as transfer fluid and kept for one minute.^7^ Sterile paper points were used to collect the transfer fluid and placed into a test tube containing 10 mL of sterile saline. This saline from each test tube was applied to nutrient agar culture plates and incubated at 37°C for 48 hours. The CFU for each plate was calculated using bacterial colony counter (Fig. 1). The bacterial count was statistically analyzed with Statistical Package for the Social Sciences software version 19, using Kruskall-Wallis analysis of variance followed by posthoc Mann-Whitney U test.

## RESULTS

The mean bacterial CFUs in the experimental groups and positive control group has been shown in Table 1. The positive control group showed the highest number of bacteria (295.40 ± 21.90), and the laser diode group showed the highest antimicrobial activity with least CFU (8.0 ± 7.87). No colonies were inoculated in the negative control, and hence, no CFU were seen in them. The mean CFUs of NaOCl group (69.80 ± 19.57) and triphala group (58.60 ± 16.63) was almost same. Better antimicrobial efficacy was seen in laser, followed by triphala and NaOCl. The comparison between the different groups has been shown in Table 2. All experimental groups showed statistically significant antimicrobial activity compared to the positive control. Laser group showed significant antimicrobial efficacy than triphala (p = 0.009) and NaOCl (p = 0.019). Triphala group showed more antibacterial activity than NaOCl, but the difference was not statistically significant.

**Table 2:** **Table 2:** Comparison of the antimicrobial efficacy of different groups

*Groups*		p value*	
Positive control *vs* NaOCl		0.009	
Positive control *vs* laser		0.009	
Positive control *vs* triphala		0.009	
NaOCl *vs* laser		0.019	
NaOCl *vs* triphala		0.341	
Laser *vs* triphala		0.009	

*Mann-Whitney U test; ^†^Sodium Hypochlorite (NaOCl)

## DISCUSSION

This randomized control trial was conducted to determine the efficacy of triphala, diode laser, and NaOCl to eradicate *E. faecalis* from the experimentally contaminated primary root canals.

*E. faecalis* is a gram positive facultative anaerobe and a known endodontic pathogen. *E. faecalis* reduces the action of lymphocytes and hence result in endodontic failure. It has serine protease, collagen-binding protein (Ace), which helps the bacteria to attach to dentin. They are also small enough to invade and survive within the dentinal tubules8 and can be isolated frequently from the failed root canal treated teeth.^2^ The efficacy of an irrigant can be demonstrated by evaluating the antimicrobial activity of the irrigant against these *E. Faecalis.*

Sodium hypochlorite has been commonly used for endodontic procedures in primary teeth. The bactericidal effect of NaOCl was due to the release of hypochlorous acid and active chlorine.^9^ The depth of penetration of NaOCl is limited to 100 μm, whereas the depth of penetration of *E. faecalis* is up to 300 to 400 μm.^10,11^

Bacteria deep in root dentin are safe from instrumentation and irrigation, due to limited depth of penetration of the irrigants. This has led to the search of new disinfectants against endodontic pathogens like *E. faecalis.* Diode laser was introduced to root canal treatment as a effective tool in disinfecting the canals.^4^ The depth of penetration of diode laser was up to 1000 μm into dentinal tubules which attributes to the superior bactericidal effect of diode laser irradiation.^10,11^ Laser irradiation with its inherent properties of local intensity enhancement, light scattering, and attenuation makes superior antimicrobial efficacy and light penetration deeper in the dentin tubules.^12,13^ The diode laser has thermal photodisruptive action in dentin, resulting in an increased bactericidal effect in the root canal system. Kuvvetli et al^14^ recommended the use of diode laser five times for 5 seconds each time, with a interval of 15 seconds between irradiations at a wavelength of 810 nm and output power of 2 W. Gutknecht et al^4^ have shown that laser had good antibacterial activity in permanent teeth. There is limited literature which assessed the antibacterial effect of lasers in primary root canals. In this study, the diode laser showed maximum antibacterial activity against *E. faecalis* in primary teeth. This was congruent with the study done by Mehrvarzfar et al,^7^ where laser had better antimicrobial activity than NaOCl and CHX. But the use of laser in a clinical setting may not be convenient considering the safety control and the cost.^15^

Natural products have been used in dental and medical fields for many years and have become more popular today. With changes in lifestyles and treatment modalities, the pathogens are also becoming more resistant. The importance of the natural medicine and herbal drugs come to play in these situations as they have an advantage of being safe, biocompatible, and nontoxic. Many herbal medicines have a potential use in endodontics. Although herbal intracanal irrigants have been used in permanent teeth, studies are limited in primary teeth.^16,17^ Triphala solution in 10% Dimethyl sulfioxide (DMSO) has good antibacterial effect and can be used as an intracanal irrigant in permanent teeth.^6^ No literature is available on the use of triphala in primary teeth. Hence, in this study, an attempt was made to find the minimum inhibitory concentration (MIC) of triphala solution. MIC of triphala in different concentrations of DMSO was evaluated. 1gm of triphala powder in 2ml of DMSO showed maximum antibacterial activity. In this concentration, the solution was too viscous to be loaded in the irrigation syringe. So in this study, triphala solution was used in 1:3 dilution. This dilution showed a slightly better antibacterial efficacy than NaOCl, but it was not statistically significant. Hence triphala solution can be used as an alternative to the conventional irrigant, NaOCl. In children, accidental exposure or overdose of NaOCl or other chemical irrigants can be harmful. Hence there is always a need for safe and natural irrigants while treating children. This in-vitro study has brought into light a) the antimicrobial efficacy of triphala solution in primary teeth b) laser has better antimicrobial efficacy than triphala and NaOCl. Further in-vivo research is required for better understanding of the efficacy of triphala as an intracanal irrigant in primary teeth.

## CONCLUSION

Within the limitations and the experimental conditions of this study, the following conclusions can be made:

- Diode laser almost completely eradicated *E. faecalis* from the root canals of primary teeth.
- Saline was not able to eradicate *E. faecalis.*
- Triphala showed better antibacterial efficacy than NaOCl. Sodium hypochlorite can be substituted with triphala due to its herbal properties.
